# Hypomineralised second primary molars: the Würzburg concept

**DOI:** 10.1007/s40368-024-00913-7

**Published:** 2024-05-28

**Authors:** K. Bekes, R. Steffen, N. Krämer

**Affiliations:** 1 Department of Paediatric Dentistry, University Clinic of Dentistry, Medical University of Vienna, 1090 Vienna, Austria; 2 Private Practice, Weinfelden, Switzerland; 3 Department of Pediatric Dentistry, Justus Liebig University, Giessen, Germany

**Keywords:** Hypomineralised second primary molars (HSPM), Molar incisor hypomineralisation (MIH), Würzburg concept, MIH treatment need index (MIH-TNI), HSPM treatment need index (HSPM-TNI), Treatment plan

## Abstract

**Purpose:**

In addition to molar incisor hypomineralisation, the occurrence of enamel hypomineralisation in the primary dentition has become increasingly important in recent years. Hypomineralised second primary molar (HSPM) is defined as hypomineralisation of systemic origin affecting from one to all four second primary molars. Some years ago, the “Würzburg concept” was introduced, which proposed a grading of MIH findings (MIH treatment need index) in combination with an appropriate treatment plan depending on the severity of the affected tooth. Recently, this concept was updated and new treatment approaches have been added. However, currently, the concept solely addresses the treatment plan for permanent teeth. As there is a need to expand its scope to encompass primary teeth and, consequently, HSPM, this paper seeks to develop the second component of the Würzburg concept, the treatment plan, for the primary dentition in response to the increased focus on the disease in recent years. Although the evidence base for the different treatment options is still weak, there is a need for guidance for clinicians in their day-to-day practice.

**Methods:**

The authors conducted a comprehensive review of the literature, encompassing clinical and laboratory studies along with published guidelines.

**Results:**

The treatment plan of the HSPM Würzburg concept contains prophylactic and regenerative aspects, non-invasive interventions, temporary and permanent restorative techniques, and extraction.

**Conclusions:**

The intention is to provide practical guidance to practitioners, acknowledging the necessity for further validation through clinical trials.

## Introduction

Enamel hypomineralisations continue to captivate the attention of the (paediatric) dental research community. They often manifest in various forms, and one specific condition is molar incisor hypomineralisation (MIH), which primarily affects the one or more first permanent molars with or without the involvement of the incisors (Weerheijm et al. 2001). In the realm of primary dentition, similar presentations are observed in the second primary molars, now acknowledged as hypomineralised second primary molars (HSPM) (Elfrink et al. 2008). HSPM is characterised by demarcated opacities, post-eruptive breakdown (PEB), atypical caries/restorations, and, in severe cases, extractions resulting from HSPM. The aetiology still remains unclear. Maternal smoking, maternal hypertension, low birth weight, prematurity, delivery complications, need for incubation, not breastfeeding, antibiotic use, fever and childhood asthma are currently discussed as potential pre-, peri- and postnatal factors (Lima et al. 2021). The average prevalence varies worldwide, but is currently estimated at 6.8% (Amend et al. 2021; McCarra et al. 2022). The condition poses a risk for MIH, exhibiting comparable clinical presentation and structural characteristics (Garot et al. 2018).

HSPM exhibits a diverse clinical presentation, presenting a substantial challenge in dental practice. The severity of enamel defects associated with HSPM varies, ranging from mild opacities with limited functional impact to more pronounced manifestations, such as extensive post-eruptive breakdown (Elfrink et al. 2008). This breakdown may also lead to sensitivity and significant discomfort for affected individuals.

For the diagnosis of HSPM, the internationally well-known and established MIH-criteria proposed by the EAPD (Lygidakis et al. 2010) can be applied (Elfrink et al. 2012; Ghanim et al. 2013). These criteria consider the specific clinical manifestations of the disease, encompassing demarcated opacities, post-eruptive enamel breakdowns, atypical restorations, and molars requiring extraction (Weerheijm 2003; Lygidakis et al. 2022; Somani et al. 2022). In addition, like MIH, hypomineralised primary teeth can be classified as mild or severe (Lygidakis et al. 2022; Somani et al. 2022).

## Development of the original MIH-Würzburg concept

During the spring conference of the German Society of Paediatric Dentistry (DGKiZ) a few years ago, the “Würzburg concept” was developed through collaboration among a working group comprising representatives from Germany, Austria, and Switzerland (Bekes et al. 2016, Bekes and Steffen 2016, Steffen et al. 2017). In 2016, this was the first MIH concept to provide both—a classification index, with regard to loss of substance and hypersensitivity, and a therapy plan based on the index. It marked a pioneering achievement in the field of MIH by introducing a classification index along with a corresponding therapeutic plan derived from the index. Since its inception, this concept has garnered increasing international recognition, as evidenced by studies conducted by several working groups (Hahn et al. 2020; Butera et al. 2022; Joshi et al. 2022; Olczak-Kowalczyk et al. 2023). The motivation behind the concept stems from the observation that the classifications prevailing in the literature at the time predominantly emphasised the dental defect as a criterion, neglecting the clinically significant aspect of combined sensitivity. Furthermore, these classifications often lacked a specific treatment recommendation (Lygidakis et al. 2010). Addressing this gap, the European Academy of Paediatric Dentistry (EAPD) updated its ‘Best Practice Guidance’ in 2022, now incorporating such a linkage (Lygidakis et al. 2022). While the “Würzburg concept” aims to assist practitioners in their daily work, it is noteworthy that the evidence supporting various treatment options remains relatively weak.

Initially, the Würzburg concept was exclusively prepared with a primary emphasis on MIH. Nevertheless, it can and should be modified to suit the specific needs of HSPM as well.

### Part 1: MIH/HSPM treatment need index (MIH-TNI/HSPM-TNI)

The MIH treatment need index (MIH-TNI) includes the presence and extent of the breakdown and the problem of hypersensitivity. It captures the clinical key symptoms associated with MIH (Bekes and Steffen 2016, Steffen et al. 2017). Accordingly, the classification encompasses four distinct grades (Table 1), which are determined based on the presence or absence of breakdown and/or hypersensitivity.

**Table 1:** HSPM treatment need index (MIH-TNI)

Index	Definition
Index 0	No HSPM, clinically sound
Index 1	HSPM: without breakdown, without hypersensitivity
Index 2	HSPM: with breakdown, without hypersensitivity
2a	Extension of defect < 1/3
2b	Extension of defect ≥ 1/3 to < 2/3
2c	Extension of defect ≥ 2/3 or/and defect close to the pulp or extraction or atypical restoration
Index 3	HSPM without breakdown, with hypersensitivity
Index 4	HSPM with breakdown, with hypersensitivity
4a	Extension of defect < 1/3
4b	Extension of defect ≥ 1/3 to < 2/3
4c	Extension of defect ≥ 2/3 or/and defect close to the pulp or extraction or atypical restoration

From a clinical perspective, HSPM exhibits similarities to MIH, characterised by enamel opacities that may lead to post-eruptive breakdown (Elfrink et al. 2008). The defects can cause significant discomfort for the child, inducing sharp pain triggered by the consumption of cold foods or exposure to cold air soon after the eruption of the affected teeth. Therefore, the TNI is applicable to all teeth—permanent and primary—and is not limited to tooth groups. It can be used in HSPM as well (HSPM treatment need index; HSPM-TNI).

### Part 2: treatment plan

The original treatment plan is presented in form of a flow chart (Bekes et al. 2016). It takes into account the different severities of MIH with the objective of assisting clinicians in their day-to-day practice. The treatment strategies encompass various aspects such as prophylaxis, regeneration, sealing, immediate treatment, and long-term planning. Recently, this treatment plan has been updated (Bekes et al. 2023). The revised management of MIH is now incorporating additional available treatment strategies and is extending its scope to encompass the treatment of hypomineralised anterior teeth.

## The HSPM Würzburg concept

The same approach can be taken for HSPM. Similar treatment approaches exist in the management of hypomineralised primary teeth: prophylaxis, regeneration, non-invasive therapy, temporary and permanent therapy as well as extraction (Elfrink and Weerheijm 2020).

**Fig. 1:**
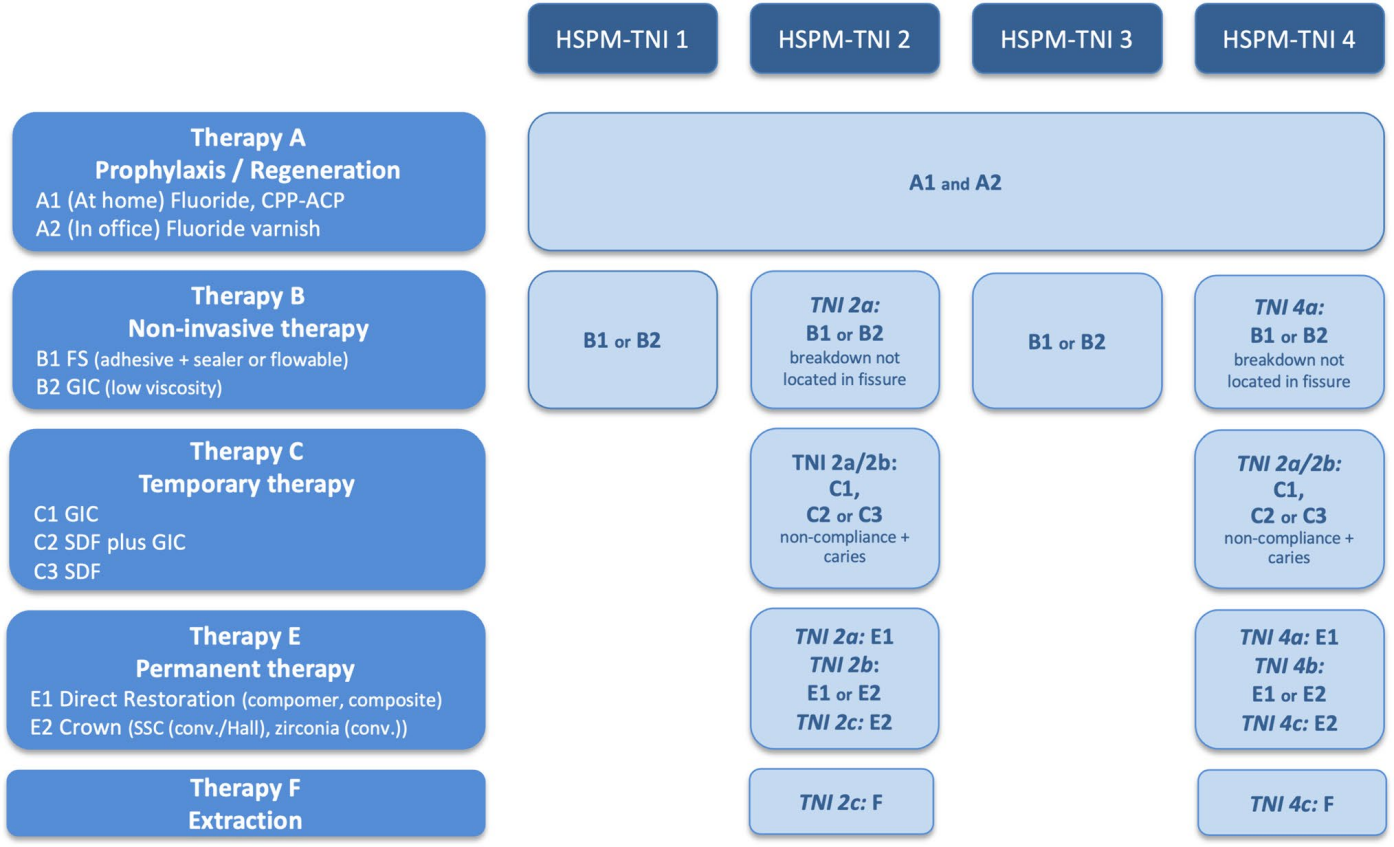
Treatment plan based on the HSPM-TNI

The structure of the flow chart is similar to the MIH-flow chart (Fig. 1): in the first horizontal row, the four indices (HSPM-TNI 1–4) are shown. In the first column, all available treatment approaches are displayed: prophylaxis (at home, in office), non-invasive therapy, temporary therapy (GIC, SDF or SDF + GIC), permanent therapy (filling or prefabricated crown) and extraction. The flow chart should be read in such a way that after the diagnosis (TNI 1–4), the user can find the treatment options in the appropriate column.

### Therapy A: prophylaxis/regeneration

Prophylaxis is not only important in MIH affected patients but also in children showing HSPM as both groups have a higher caries risk (Lygidakis et al. 2022; Ben Salem et al. 2023). Higher prevalences of dental caries in MIH/HSPM children compared with non-affected ones were found by Ben Salem et al. in a recent review (Ben Salem et al. 2023). Therefore, prophylaxis should be performed directly after hypomineralised teeth eruption independently of the severity of the diagnosed TNI. Fluoridated tooth pastes should be used twice a day at home. Topical fluoride varnish can be applied ‘in office’ 2–4 times per year depending on caries risk (Toumba et al. 2019). The additional use of mineral containing agents (e.g. calcium glycerophosphate (CaGP), casein phosphopeptide amorphous calcium phosphate (CPP-ACP), or casein phosphopeptide amorphous calcium fluoride phosphate (CPP-ACFP)) are recommended to hypomineralised lesions with mineral deposition (Sezer and Kargul 2022).

### Therapy B: non-invasive therapy

Non-invasive therapy strategies include sealings. One possibility is the use of a sealant or flowable in combination with an adhesive if the tooth is fully erupted and the child is compliant. Another option is the application of a glass ionomer cement (if the tooth is not fully erupted or if there is a non-compliance).

### Therapy C: temporary therapy

The original flow chart for MIH includes two temporary therapy options: short-term (therapy C) and long-term (therapy D). Regarding HSPM, no subdivision is needed. Therefore, this flow chart only includes therapy C. First, temporary treatment approaches include the application of a glass ionomer cement in case of defects (dimension < 2/3 of the surface) being present. Glass ionomer cements that are less technique-sensitive and can be placed in only one increment, favouring clinical management (Amend et al. 2022). If caries is present and the child is non-compliant SDF without/with GIC might be considered as treatment of choice (BaniHani et al. 2022).

### Therapy E: permanent therapy

The following treatment options can be considered definitive for the restoration of primary teeth: restorations using composite or compomer materials and preformed crowns (stainless steel or zirconia) (Amend et al. 2022). When applying crowns, a distinction can also be made between the conventional approach and the Hall technique (BaniHani et al. 2022; Hu et al. 2022).

### Therapy F: extraction

The therapy plan is finalised with therapy F, extraction. In severe cases, when primary molars show severe post-eruptive breakdowns, the pulp is involved or dental abscesses are present, extraction should be the treatment of choice in HSPM. The indications are similar to the management of MIH (Lygidakis et al. 2022).

#### HSPM-TNI 1

For hypomineralised primary teeth that do not show any post-eruptive breakdowns or hypersensitivity (HSPM-TNI 1), prophylaxis/regeneration is the starting point. In addition to this, the sealing of the fissures could be an option. If the tooth is fully erupted, this procedure should be carried out with a conventional fissure sealant or a flowable with the pre-application of an adhesive (Lygidakis et al. 2009). If the molar has not yet fully erupted, a temporary fissure sealant should be applied using a low viscosity glass ionomer cement.

#### HSPM-TNI 2

A TNI 2 is defined as substance loss being present and hypersensitivity being absent. If the breakdown is not located in the fissure and involves < 1/3 of the surface of the tooth, sealing therapy (B) may be a first step in the management of HSPM beside prophylaxis. If the breakdown is found in the fissure, the defect is up to 2/3 or close to the pulp, and the child is not compliant, a temporary therapy (C) using a GIC is the option of choice. If, in addition to non-compliance, caries is also present, SDF can be used with or without GIC (C2, C3) (Zaffarano et al. 2022, Inchingolo et al. 2023). Permanent restorations (E) include the use of composite or compomer-based fillings (Amend et al. 2022). Alternatively, a prefabricated (preformed) stainless steel crowns can be chosen (D) (Declerck and Mampay 2021; Amend et al. 2022). The preparation technique can be conventional (both materials) or Hall (stainless steel crown, (Innes et al. 2007)). For larger defects (TNI 2c), especially where there is a risk of compromising pulp integrity, prefabricated zirconia crowns may be an option for restoration. However, due to the large loss of substance during preparation for this restoration, there is a high risk of artificial pulp opening in less severe cases (Mohn et al. 2022, Sparks et al. 2022). In severe cases (TNI 2c), extraction is also an option.

#### HSPM-TNI 3

Hypersensitive hypomineralised primary molars with no breakdown can be sealed. Thereby, resin-based sealants in combination with an adhesive can be applied similar to the treatment of MIH (Lygidakis et al. 2022). If the tooth has not fully erupted and the child is not cooperative, sealing with a low viscosity glass ionomer cement (B2) can also be performed.

#### HSPM-TNI 4

In the case of the presence of a substance loss and hypersensitivity, the treatment plan is similar to the options presented for the TNI 2. The size and location of the defect is important. Minimal breakdowns that do not occur in the fissure can be sealed. Temporary therapy options include the application of GIC when defects can be found in the fissure or if the breakdown is > 1/3 or > 2/3 in its extension or close to the pulp. In case of non-compliance and caries being present, the application of SDF without/with GIC should be considered (Zaffarano et al. 2022, Inchingolo et al. 2023). Permanent restorations (E) comprise the use of composite/compomer fillings or of prefabricated crowns (stainless steel or zircona) (Amend et al. 2022). Extraction marks the end of increasingly invasive methods and must be considered in severe cases with breakdowns of more than 2/3 of the crown.

## Discussion

HSPMs exhibit vulnerability to comparable issues observed in teeth affected by MIH. These include heightened susceptibility to the formation of cavities, sensitivity, and an elevated requirement for dental treatments such as restorations and extractions (Owen et al. 2018). For this reason, it makes sense to adapt proven classification systems and therapeutic approaches. This has already been realised in recent years with the adoption of the EAPD diagnostic criteria for MIH in an adapted form (Owen et al. 2018; Lima et al. 2020; Singh et al. 2020).

This paper is the first approach to provide a treatment concept for hypomineralised primary teeth including a classification (HSPM-TNI) for affected teeth and corresponding treatment options. This guidance can aid practitioners in their daily practice, although the evidence supporting various treatment options in HSPM currently remains weak and further studies are needed.

## Conclusions

The clinical picture of hypomineralised second primary molars and the resulting necessary treatment procedures can be very variable. The “Würzburg concepts” which was originally developed for teeth affected by MIH can also be applied in primary teeth. Part 1 of the concept, the treatment need index (TNI) can be adopted without change. Part 2, the treatment plan, has been has been adjusted to the needs of the primary dentition. The proof of concept should be demonstrated in further clinical trials.
